# An AIDS patient with urine retention

**DOI:** 10.1186/s12879-019-4641-8

**Published:** 2019-12-12

**Authors:** Lijun Xu, Ran Tao, Qiyu Zhao, Jinlin Cheng, Biao Zhu

**Affiliations:** 1 National Clinical Research Center for Infectious Diseases, the First Affiliated Hospital, College of Medicine, Zhejiang University, Qingchun Rd, Hangzhou, China; 2 The State Key Laboratory for Diagnosis and Treatment of Infectious Diseases, the First Affiliated Hospital, College of Medicine, Zhejiang University, No.79, Qingchun Rd, Hangzhou City, 310003 China; 3 Ultrasonography department, the First Affiliated Hospital, College of Medicine, Zhejiang University, Qingchun Rd, Hangzhou, China

**Keywords:** Cryptococcal prostatitis, HIV, Tuberculosis

## Abstract

**Background:**

Cryptococcal prostatitis is a rare clinical disease and has never been reported in China.

**Case presentation:**

We report on a male HIV-infected patient with pulmonary and prostate cryptococcosis that was misdiagnosed (as tuberculosis) and delayed diagnosed. Although the patients accepted anti-fungal treatment and anti-retroviral treatment finally, the physician’s mistakes reflect the rarity of this condition in China.

**Conclusion:**

Cryptococcal prostatitis is a rare disease that unusually presents in immunodeficient patients. Physicians should have a heightened awareness of this particular infection in the immunodeficient population.

## Background

Cryptococcosis is a common opportunistic infection in HIV-infected patients, causing up to 70% mortality at 3 months in some areas [1]. Patients with HIV tend to have more extraneural involvement and cryptococcemia than those without HIV [2]. Therefore, being aware of atypical *Cryptococcus* infection sites is critical when caring for HIV patients.

Cryptococcal prostatitis is rare. It usually presents in immunocompromised patients, such as those with diabetes, liver cirrhosis or organ transplantation [3–6]. *Cryptococcus*-infected prostates are occasionally reported in patients with AIDS [7, 8]. However, cryptococcal prostatitis has never been reported in China. Here, we report the first Chinese case of a patient with AIDS and urinary retention, in which the infection was misdiagnosed as tuberculosis before being correctly diagnosed as cryptococcal prostatitis.

## Case presentation

In October 2017, a 47-year-old man had presented to a local hospital with cough and weight loss. He tested positive for HIV and a chest CT scan indicated a cavity in the right upper pulmonary lobe (Additional file 1). His CD4^+^ T cell count was 78 cells/μl and his sputum smear was negative for tuberculosis. He was diagnosed as HIV positive with tuberculosis infection (smear negative) and started antiviral therapy with tenofovir, lamivudine and efavirenz after 2 weeks of anti-tuberculosis treatment with isoniazid, rifampin, ethambutol and pyrazinamide.

**Fig. 1:**
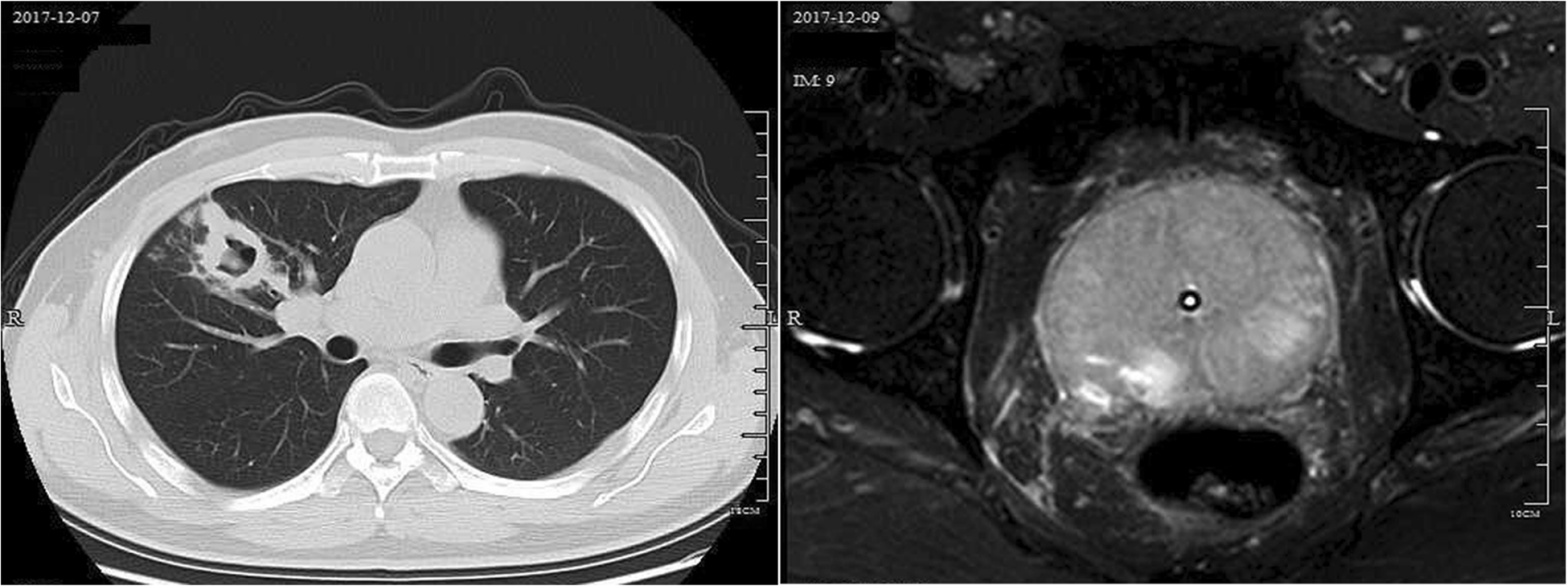
Radiology images of patient. **a**. Chest CT in Dec 2017 indicated a cavity with effusion in the upper right lung; **b**. Enhanced MRI of the prostate in Dec 2017 revealed an abnormal patchy signal in the central gland of the prostate

**Fig. 2:**
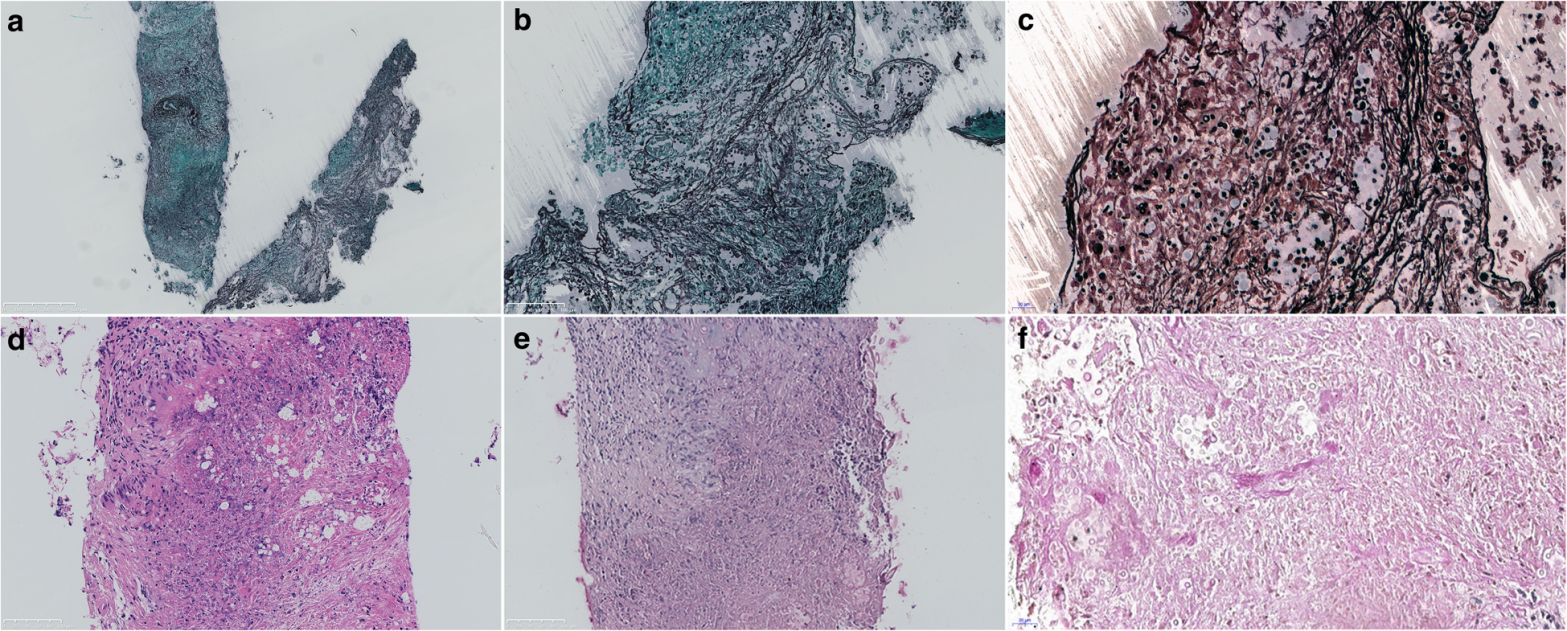
Pathological finding in Prostate biopsy. Cryptococcal capsules were shown in histiocytes. **a**. Gomori-methenamine silver (GSM) staining (50×); **b**. GSM staining (200×);c. GSM staining (400×):**d**. Hematoxylin-Eosin (HE) staining (200×); **d**. Periodic Acid–Schiff (PAS) staining (200×); **f**. PAS staining (400×)

In December 2017, the patient presented to the same hospital after having urinary retention for 2 weeks. He had no other symptoms such as headache, fever or cough. His prostate was found to be enlarged, and the doctor suggested continuing anti-tuberculosis treatment and inserted a urinary catheter to relieve the patient’s urinary retention. Given that the urinary retention had deteriorated for two weeks before this visiting, the doctor ***s***uggested the patient attend the First Affiliated Hospital of Zhejiang University for better solution. After admission, physical examination revealed no significant clinical findings except for the presence of the urinary catheter. The sputum culture, smear and interferon-γ release assay were all negative for *Mycobacterium tuberculosis*. Lumbar puncture indicated a nearly normal cerebrospinal fluid (CSF) profile and the CSF was negative for cryptococcal antigen, so urinary retention as a result of central nervous system infection seemed unlikely. A CT scan indicated a cavity with effusion in the upper right lung (Fig. 1.a). Bronchoscopy was performed, and a lateral flow immunoassay and culture of the bronchoalveolar lavage fluid (BALF) were both positive for *Cryptococcus neoformans*. Enhanced MRI of the prostate revealed an abnormal patchy signal in the central gland of the prostate that was isointense on T1-weighted imaging (WI), high intensity on T2WI, and no signal on diffusion-weighted imaging. The signal of T2 W1 was reduced in the seminal vesicles and peripheral zone of the prostate, and T1 W1 in the peripheral zone was enhanced (Fig. 1. b). An haematoxylin and eosin (H&E) stain of the ultrasound-guided prostatic biopsy showed granulomatous inflammation; periodic acid–Schiff staining of the biopsied tissue was positive, Mayer’s Mucicarmine stain was negative, Gomori-methenamine silver staining was positive, and Ziehl-Neelsen staining was negative (Fig. 2). Culture of the prostatic tissue grew *Cryptococcus neoformans*. The patient was diagnosed with AIDS with pulmonary cryptococcosis and cryptococcal prostatitis. Oral voriconazole (200 mg bid) combined with oral flucytosine (100 mg/kg/d) for 2 months and then oral fluconazole (600 mg/d) for maintenance treatment for 4 months that relieved the cryptococcal prostatitis and urinary retention (Additional file 2).

## Discussion and conclusions

China has a high prevalence of tuberculosis infection, and tuberculosis is a common opportunistic infection in Chinese HIV-infected patients. Smear-negative tuberculosis is more common in patients with HIV infection than in those without [9, 10]. However, there have been reports of *Cryptococcus* infection in immunocompromised patients with atypical presentations being misdiagnosed as tuberculosis [11, 12].

Our patient was misdiagnosed as having smear-negative pulmonary tuberculosis. *Cryptococcus* infection was hinted at by the lung cavity and the prostate hypertrophy; this was then confirmed by the presence of *Cryptococcus* in the BALF and prostatic biopsy specimen.

Although the correct diagnosis was reached eventually, this case provides some valuable lessons. First, a detailed pathogen screen should be done in HIV patients, especially in those with pneumonia; it is sometimes difficult to make accurate diagnoses based only on radiology. A lateral flow immunoassay for cryptococcal antigen is a rapid and reliable diagnosis method for suspected *Cryptococcus* infection [13]. Second, prostate infections should be a ‘red flag’ for urologists and pathologists in general hospitals in China. There are two types of hospitals in China responsible for HIV treatment; specialized and general hospitals. Doctors in some general hospitals, especially in those without HIV/AIDS wards, are less aware of atypical infections in HIV patients. Strengthening and highlighting AIDS-associated medical knowledge in these practitioners is important. Third**,** cryptococcal prostatitis is an uncommon disease in clinical practice, which may influence some doctors’ correctly diagnosis and treatment decisions in some circumstances.

In summary, cryptococcal prostatitis is a rare disease that unusually presents in immunodeficient patients. Physicians should have a heightened awareness of this particular infection in the immunodeficient population.

## Supplementary information

**Additional file 1.** Chest CT in Oct 2017.

**Additional file 2.** Prostate CT after 6 month of anti-fungal treatment.

## Data Availability

Some original images were provided as supplements.

## Abbreviations

AIDS: Acquired immune deficient syndrome
BLAF: Bronchoalveolar lavage fluid
CSF: Cerebrospinal fluid
CT: Computed tomography
HIV: Human immunodeficiency virus
MRI: Magnetic Resonance Imaging
