# Supplementary figures and images for: An AIDS patient with urine retention
Source: BMC Infect Dis. 2019 Dec 12;19:1051. doi: 10.1186/s12879-019-4641-8 (PMC6909583; doi:10.1186/s12879-019-4641-8)

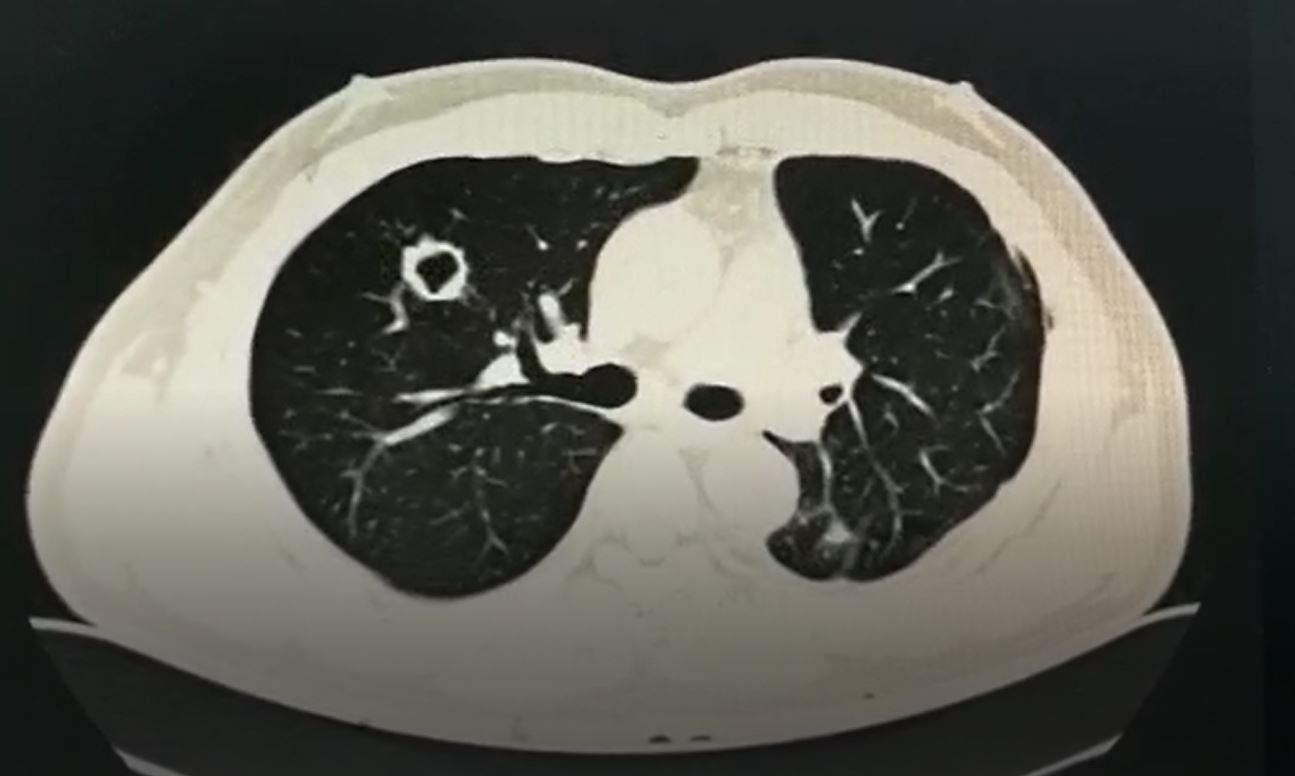

Supplement: Supplementary file 1 — Additional file 1. Chest CT in Oct 2017. [file 12879_2019_4641_MOESM1_ESM.jpg]

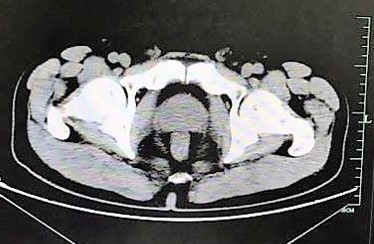

Supplement: Supplementary file 2 — Additional file 2. Prostate CT after 6 month of anti-fungal treatment. [file 12879_2019_4641_MOESM2_ESM.jpg]
